# Senescent vs. non-senescent cells in the human annulus in vivo: Cell harvest with laser capture microdissection and gene expression studies with microarray analysis

**DOI:** 10.1186/1472-6750-10-5

**Published:** 2010-01-28

**Authors:** Helen E Gruber, Gretchen L Hoelscher, Jane A Ingram, Natalia Zinchenko, Edward N Hanley

**Affiliations:** 1 Department of Orthopaedic Surgery, Carolinas Medical Center, Charlotte, N.C., USA

## Abstract

**Background:**

Senescent cells are well-recognized in the aging/degenerating human disc. Senescent cells are viable, cannot divide, remain metabolically active and accumulate within the disc over time. Molecular analysis of senescent cells in tissue offers a special challenge since there are no cell surface markers for senescence which would let one use fluorescence-activated cell sorting as a method for separating out senescent cells.

**Methods:**

We employed a novel laser capture microdissection (LCM) design to selectively harvest senescent and non-senescent annulus cells in paraffin-embedded tissue, and compared their gene expression with microarray analysis. LCM was used to separately harvest senescent and non-senescent cells from 11 human annulus specimens.

**Results:**

Microarray analysis revealed significant differences in expression levels in senescent cells vs non-senescent cells: 292 genes were upregulated, and 321 downregulated. Genes with established relationships to senescence were found to be significantly upregulated in senescent cells vs. non-senescent cells: p38 (MPAK14), RB-Associated KRAB zinc finger, Discoidin, CUB and LCCL domain, growth arrest and DNA-damage inducible beta, p28ING5, sphingosine-1-phosphate receptor 2 and somatostatin receptor 3; cyclin-dependent kinase 8 showed significant downregulation in senescent cells. Nitric oxidase synthase 1, and heat shock 70 kDa protein 6, both of which were significantly down-regulated in senescent cells, also showed significant changes. Additional genes related to cytokines, cell proliferation, and other processes were also identified.

**Conclusions:**

Our LCM-microarray analyses identified a set of genes associated with senescence which were significantly upregulated in senescent vs non-senescent cells in the human annulus. These genes include p38 MAP kinase, discoidin, inhibitor of growth family member 5, and growth arrest and DNA-damage-inducible beta. Other genes, including genes associated with cell proliferation, extracellular matrix formation, cell signaling and other cell functions also showed significant modulation in senescent vs non-senescent cells. The aging/degenerating disc undergoes a well-recognized loss of cells; understanding senescent cells is important since their presence further reduces the disc's ability to generate new cells to replace those lost to necrosis or apoptosis.

## Background

Cell senescence (also termed replicative senescence) occurs when normal cells stop dividing. This phenomenon was initially described more than 40 years ago during studies of cultured human fibroblasts [1]. Senescent cells are viable, but exhibit alterations in phenotype and altered gene expression patterns [2-5]. Senescent cells may have altered responsiveness to external stimuli and may secrete factors which can influence neighboring cells or their nearby extracellular matrix (ECM). There is currently a great deal of interest in the manner in which cell senescence may contribute to age-associated loss of function or age-related pathology in vivo, and molecular studies are directed towards elucidating mechanisms and pathways which activate the senescence program in cells [6].

The current views of cell senescence not only recognize that it is a condition in which cells can no longer respond to mitogenic signals and thus cannot proliferate, but also point out that senescence also is associated with alterations in nuclear structure, protein processing, gene expression and cell metabolism. The senescent state is a complex response to specific trigger(s) or multiple signaling pathways, including telomere uncapping, oxidative stress, DNA damage and oncogene activation [3,7,8]. Senescence represents a general cellular response mechanism which, when activated, results in numerous morphologic and functional changes [2]. There is currently no one single marker for senescent cells, but researchers now have characterized a number of important characteristics which have been summarized by Campisi and d'Adda di Faggana [9] and Cichowski and Hahn [10].

Microarray analysis, which we used in the present work, has been shown to be a powerful analytical tool in previous studies of cell senescence in studies of cultured cells [11]. Shelton et al. studied senescence in three cell types, dermal fibroblasts, retinal pigment epithelial cells, and vascular endothelial cells [12], and Zhang et al. examined senescent fibroblasts and mammary epithelial cells [13]. These studies, and gene expression profiling studies [14], showed that specific cell types have specific patterns of up- or down-regulation of gene expression during senescence.

In the aging intervertebral disc, there is a well-recognized loss of cells, which puts the remaining cell population at risk for any diminution in cell function. A number of years ago, Buckwalter provided insightful comments which pointed to the need to learn more about this process which blocks future cell division capability in the disc and alters the cell's functional capacity [15,16].

A number of major studies have now verified the presence of senescent cells in the aging/degenerating human disc. Studies by Roberts et al. have provided evidence that there was a greater proportion of senescent cells in herniated than non-herniated discs, and more senescent cells in the nucleus pulposus compared to the annulus [17]. Work by Le Maitre et al. showed that the senescent cell phenotype is associated with increased catabolism involving metalloproteinase 13 (MMP 13) and aggrecanase (ADAMTS 5) [18]. This finding was important because it links senescence with matrix degradation, one of the major problems in disc degeneration. This study also showed that disc cells exhibit accelerated senescence with decreased telomere length.

Studies from our laboratory have shown that the proportion of senescent cells increased significantly with increasing stages of disc degeneration (p < 0.0001) [19]. Another publication from our lab has provided additional information on disc cell senescence. Using laser capture microdissection and microarray analysis, this study (described in more detail below) identified two senescence-related genes which were significantly up-regulated in more degenerated discs compared to healthier discs [20]: growth arrest-specific 1 gene (GAS) (which inhibits DNA synthesis, inhibits cell cycle progression in vitro, and is expressed in senescent fibroblasts [21-23]. The second significantly upregulated gene was lysyl oxidase-like 2 (LOXL2), which has been seen to be expressed in senescent human fibroblasts [24,25]. More recently, we have also shown that increased cell senescence is associated in vivo with decreased cell proliferation in the degenerating annulus [26].

Since senescent cells cannot divide, they may reduce the disc's ability to generate new cells to replace those lost to necrosis or apoptosis. Senescent cells also accumulate over time, and their metabolic products may contribute to pathologic changes seen in degenerating discs. Because of the importance of senescence, in the present study we utilize laser capture microdissection (LCM) to specifically harvest senescent cells from the annulus, determine gene expression patterns using microarray analysis, and then compare and contrast the senescent cell expression patterns with patterns from paired non-senescent cells harvested from the same histologic section.

## Methods

### Clinical Study Population

Experimental study of human disc specimens was approved prospectively by the authors' Human Subjects Institutional Review Board at Carolinas Medical Center. The need for informed consent was waived since disc tissue was removed as part of routine surgical practice. Scoring of disc degeneration utilized the Thompson scoring system; this system scores disc degeneration over the spectrum from a healthy disc (Thompson grade I) to discs with advanced degeneration (grade V, the most advanced stage of degeneration) [27]. Patient specimens were derived from surgical disc procedures performed on individuals with herniated discs and degenerative disc disease. Surgical specimens were transported to the laboratory in sterile tissue culture medium.

#### Disc Specimens

Table 1 presents the subject demographic features for specimens utilized in this study. Specimens were graded using the Thompson scoring system [27] where Grade I describes a healthy disc with abundant proteoglycan and normal collagen lamellar structure; grades progress up to Grade V which denotes a severely degenerated disc with prominent collagen lamellar bundles and decreased proteoglycan.

**Table 1:** Demographic Features of Subjects and Percentages of Senescent Cells *

Subject Number	% Senescent Cells **	Age (years)	Gender	Site	Thompson Grade
1	68.1	28	Female	L3-L4	3

2	76.4	46	Female	L4-L5	4

3	71.8	56	Male	L4-L5	4

4	74.5	45	Male	L5-S1	4

5	80.2	54	Female	C4-C5	4

6	92.0	32	Male	L5-S1	4

7	64.4	56	Male	L4-L5	5

8	68.6	37	Male	L4-L5	3.5

9	57.1	57	Male	L4-L5	3

10	46.1	26	Female	L5-S1	3

11	51.8	37	Male	L5-S1	3

* * L, lumbar; S, sacral; C, cervical.

** Determined from measurement of senescent cells (using SA-β-gal immunohistochemistry) on sections adjacent to those used for LCM.

#### Study Design

In the present study we utilize LCM to specifically harvest senescent cells from the annulus, determine gene expression patterns using microarray analysis, and then compare and contrast the senescent cell expression patterns with patterns from paired non-senescent cells harvested from the same histologic section.

### Comparison of Histochemical Senescent Cell Staining with the Technique Used for Senescence-Associated-β-galactosidase Immunolocalization for Laser Capture Micro-dissection

The traditional histochemical staining method which is utilized routinely for identification of senescent cultured cells unfortunately will not work on paraffin-embedded tissue. Therefore, we carried out an initial study to confirm that the immunohistochemical method to be used with LCM produced results which were not statistically different from those seen with the histochemical staining procedure. For this study, we used cultured disc cells from four surgical patients; annulus cells were derived and passaged as previously described [28], and assessed using a stress-induced in vitro model recently developed in our lab [29]. Briefly, cells are plated on multichamber slides (Lab-Tek™ Chamber Slide™ System (Nunc, Napierville, IL)), allowed to attach, and are then exposed to either control conditions or H_2_O_2 _exposure as a method of exogenous production of stress-induced premature cell senescence. Cells are exposed to 50 μM H_2_O_2 _for 2 hours, rinsed, and allowed to grow for 3 days. At termination, cells are rinsed, fixed with 10% neutral-buffered formalin for 10 min, and stored in 70% ethanol until processed for either histochemical localization of localization of senescence, or immunohistochemical detection of senescence as described above.

Histochemical identification of senescent cells was performed using the Senescent Cells Staining Kit (Sigma, St Louis, MO) according to manufacturer's instructions. All reagents were provided in the kit. Cells were washed two times in PBS and fixed in the fixative solution for 7 minutes at room temperature. Cells were then rinsed three times in PBS and then incubated in the staining solution overnight at 37°C. Cells were rinsed in PBS and counterstained with Nuclear Fast Red (Sigma) for 5 minutes.

The percentage of senescent cells was determined for each of four separate cultures of human annulus cells in both control and H_2_O_2_-treated cultures.

### Senescence-Associated-β-galactosidase Immunolocalization for Laser Capture Microdissection

Specimens were fixed overnight in 10% neutral buffered formalin (Allegiance, McGaw Park, IL), and then transferred to 70% Ethyl alcohol (AAPER, Shelbyville, KY) for holding until processed for paraffin embedding. Specimens were processed on TBS ATP1 Tissue Processor (TBS, Durham, NC), and embedded in Paraplast Plus (ThermoShandon, Pittsburgh, PA) paraffin. 4 μm sections were cut with a Leica (Nussloch, Germany) RM2025 microtome using RNase-free techniques and mounted on Superfrost Slides (Allegiance). Slides were cut the day they were to be processed for immunohistochemistry, and placed in 60°C oven for 30 minutes. Slides were deparaffinized using the reagents provided in the Paradise Reagent System (Molecular Devices, Sunnyvale, CA).

The immunofluorescence procedure utilized the Histogene LCM Immunofluorescence Staining Kit (Molecular Devices). The primary antibody; anti-β-galactosidase (Promega, Madison, WI) was used at a dilution of 1:20 for 10 minutes. Biotinylated Link Antibody (Dako, Carpenteria, CA) was applied for 5 minutes followed by Cy3 Streptavidin (provided in kit). All steps were performed at 4°C. Slides were dehydrated using reagents and protocol provided in Histogene Kit, air dried for 5 minutes, and laser capture microdissection performed as described below.

### Laser Capture Microdissection

LCM was carried out using the Arcturus PixCell lle LCM1106. Cells were harvested using standard LCM techniques as previously described [20]. Histologic sections adjacent to those used for LCM were first examined to ensure that only annulus regions were present. During LCM, a special film attached to a microfuge cap was placed on top of the section. Cells of interest were selected for laser removal and marked by circles. When all cells had been selected, a finely focused laser pulse was used to melt the film and allow cells to be harvested when the cap is removed. Senescent cells were selected and captured first. Remaining non-senescent cells were then scraped from the histology slide. Senescent and non-senescent cells were then processed for microarray analysis as described below.

### Microarray Analysis

Samples were prepared per instructions in the Paradise Reagent System (Arcturus Bioscience, Inc., Mountain View, CA) for steps 1 to 4 and the Affymetrix GeneChip Expression Analysis Technical Manual (copyright 2002, Affymetrix, Inc., Rev. 3, Part number 701021) for steps 5 and later. Briefly, the major steps were: 1) Total RNA was extracted from the isolated cells; 2) The total extracted RNA was converted to double-stranded cDNA; 3) cDNA was expressed as cRNA by *in vitro *transcription; 4) cRNA was used to prime a second round of cDNA synthesis; 5) The second-round cDNA was expressed as biotin-labeled cRNA with the Affymetrix 3' Amplification Reagents for IVT Labeling (Affymetrix, P/N 900449); 6) Biotin-labeled cRNA was fragmented non-enzymatically.

The Affymetrix human U133 X3P array, with probes for 47,000 human transcripts with all probe sets within 300 base pairs of the 3' end of the transcript, was used in this study since this specialized design permits the quantification of fragmented RNA from paraffin-embedded tissue. The GCOS Affymetrix GeneChip Operating System (version 1.2, Affymetrix, Santa Clara, CA) was used for determining gene expression levels.

Microarray data used in the present study can be viewed in the study named GSE17077 study at the following website: http://www.ncbi.nlm.nih.gov/geo/query/acc.cgi?acc=GSE17077.

### Statistical Analyses

GeneSifter™ web-based software (VizX Labs, Seattle, WA, USA; http://www.genesifter.net) was used to analyze all microarray data. Using GC-RMA (Robust multi-array average), Affymetrix '.cel' files were uploaded to the GeneSifter™ web site and normalized. Using the student t-test (2 tailed, unpaired), statistical significance was determined (p < 0.05). The fold change was set at 1.02. Gene Ontologies (GO) were generated by GeneSifter™ based on the Gene Ontology Consortium (http://www.geneontology.org/GO.doc.html[30]).

## Results

### Identification of Senescent Cells Using Senescence-Associated-β-galactosidase Immunolocalization Does Not Significantly Differ from Identification Using Histochemical Senescence Detection

**Figure 1:**
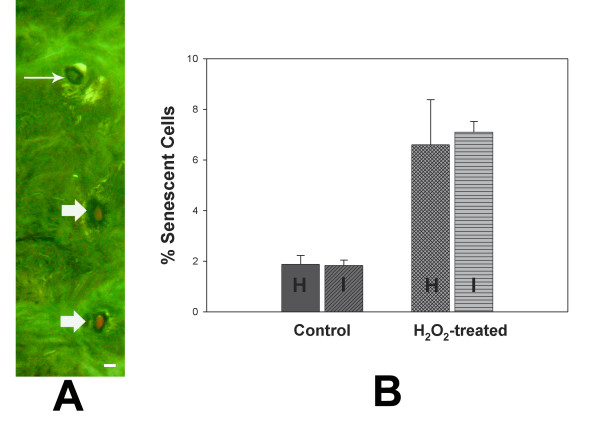
**A: Localization of senescent cells (red; bold arrows) and non-senescent cells (thin arrows) using immunofluorescent localization of senescent-associated-β-galactosidase in paraffin-embedded human annulus tissue**. (Bar = 10 μm). B: No significant difference was identified in vitro when control or H_2_O_2_-treated annulus cells were quantified using histochemical (H) vs. the immunofluoresce (I) method to detect senescent cells.

In the experimental design utilized here, LCM was employed to separately harvest senescent and non-senescent cells from paraffin sections of human disc tissue. The traditional histochemical pH 6.0 staining method routinely for identification of senescent cultured cells [31,32] unfortunately does not work on paraffin-embedded tissue. Therefore, senescent cells were identified here based on immunofluorescent localization of senescence-Associated-β-galactosidase (Figure 1A). We carried out an initial study to confirm that the immunohistochemical method for identification of senescent cells did not statistically differ from the histochemical staining of senescent cells.

This analysis required use of cultured cells. Annulus cells were cultured from four surgical disc specimens as previously described [28], cells expanded, and cultured on multi chamber slides. Replicate cultures of control cells, and cells induced into senescence via oxidative stress [29], were then assessed using the histochemical method or the immunohistochemical methodology involving localization of senescence-associated-β-galactosidase [19]. There was no statistically significant difference in the % of senescent cells in our in vitro study for either control cells, or cells induced into premature stress-induced senescence by H_2_O_2 _exposure (Figure 1B). This in vitro work validated our use of the immunocytochemistry to localize senescent cells for LCM harvest and subsequent microarray analysis.

### Clinical Study Population

Eleven annulus specimens, Thompson grade 3-4, were utilized to harvest senescent and non-senescent cells from the human annulus. Table 1 presents the demographic features of the subjects, and the percentages of senescent cells, in the discs evaluated here.

### Microarray Analysis

Analysis of genes with significant differences in expression levels in senescent cells vs non-senescent cells showed that 292 genes were upregulated, and 321 downregulated. We further analyzed expression patterns using ontology analyses for genes involved in cell proliferation, in ECM formation and in ECM degradation, cell adhesion, cell signaling, apoptosis, and genes related to cytokines/inflammation. Microarray data used in the present study can be viewed in the study named GSE17077 study at the following website: http://www.ncbi.nlm.nih.gov/geo/query/acc.cgi?acc=GSE17077. Major findings are listed below.

### Genes Related to Cell Senescence or Cell Proliferation with Significant Expression Differences in Senescent vs. Non-Senescent Annulus Cells

One major focus of our gene analysis centered upon genes known to have a previously established role in cell senescence (Table 2).

**Table 2:** Senescence-Related Genes with Significant Differences in Gene Expression Levels in Senescent vs. Non-Senescent Annulus Cells

Gene Name	Ratio/Fold Change	Direction	P value	Gene Identifier
RB-associated KRAB zinc finger	1.51	Up	0.022	AW138835

Discoidin, CUB and LCCL domain containing protein 2	1.47	Up	0.038	AW300360

p38 (p38 MAP kinase; MPAK14)	1.37	Up	0.029	AF218033

Inhibitor of growth family, member 5 (p28ING5)	1.18	Up	0.046	BC005370

Growth arrest and DNA-damage-inducible, beta	1.06	Up	0.040	AV658684

Somatostatin receptor 3	1.03	Up	0.048	NM_001051

Interferon induced transmembrane protein 1 (9-27)	1.08	Up	0.038	NM_003641

Sphingosine-1-phosphate receptor 2	1.38	Up	0.046	NM_004230

Cyclin-dependent kinase 8 (CDK8)	1.12	Down	0.049	NM_001260

Nitric oxide synthase 1	1.06	Down	0.017	U31466

Heat shock 70 kDa protein 6 (HSP70B)	1.11	Down	0.020	X51757

Several genes related to senescence were found to be significantly upregulated in senescent cells vs. non-senescent cells: p38 (MPAK14), RB-Associated KRAB zinc finger, Discoidin, CUB and LCCL domain, growth arrest and DNA-damage inducible beta, inhibitor of growth family member 5 (p28ING5), sphingosine-1-phosphate receptor 2 and somatostatin receptor 3. Another known gene related to senescence, cyclin-dependent kinase 8, showed significant downregulation in senescent cells. Nitric oxidase synthase 1, and heat shock 70 kDa protein 6, both of which were significantly down-regulated in senescent cells, also showed significant changes.

A number of genes related to the cell cycle or cell proliferation were identified which showed significant differences in senescent vs. non-senescent cells (Table 3). Significantly upregulated genes included bone morphogenetic protein receptor, type II (serine/threonine kinase), and protein tyrosine phosphatase, receptor type A. Several significantly downregulated genes were also found to be present in the senescent cells; these included alpha-2-glycoprotein 1, tumor necrosis factor superfamily, member 13b, integrin-linked kinase-2, the G1 to S phase transition 2 gene, cell division cycle 2-like 6 (CDK8-like) gene, and Ras homolog gene family member H.

**Table 3:** Cell Proliferation or Cell Cycle Genes with Significant Expression Differences in Senescent vs. Non-Senescent Annulus Cells

Gene Name	Ratio/Fold Change	Direction	P value	Gene Identifier
Protein tyrosine phosphatase, receptor type, A	1.68	Up	0.043	BF740139

Kallikrein-related peptidase 4	1.44	Up	0.025	AF113140

Leucine zipper, putative tumor suppressor 1	1.35	Up	0.033	BE312985

Leucine zipper, putative tumor suppressor 1	1.35	Up	0.033	BE312985

SPEG complex locus	1.26	Up	0.040	AL512705

KIAA1009	1.25	Up	0.042	NM_014895

Bone morphogenetic protein receptor, type II (serine/threonine kinase)	1.15	Up	0.023	U20165

Pyrin and HIN domain family, member 1	1.58	Down	0.005	AK024890

Alpha-2-glycoprotein 1, zinc-binding	1.23	Down	0.041	D90427

Arachidonate 15-lipoxygenase	1.21	Down	0.040	NM_001140

Ras homolog gene family, member H	1.17	Down	0.046	NM_004310

Cell division cycle 2-like 6 (CDK8-like)	1.16	Down	0.016	AA994004


Tumor necrosis factor (ligand) superfamily, member 13b	1.13	Down	0.024	AF134715

Triple functional domain (PTPRF interacting)	1.13	Down	0.027	BF223718

Sialophorin (leukosialin, CD43)	1.07	Down	0.042	J04168

G1 to S phase transition 2	1.04	Down	0.018	NM_018094

### Other Significant Gene Expression Differences in Senescent vs Non-senescent Annulus Cells

Because senescent cells remain metabolically active even through they can no longer divide, we were also interested in other gene expression patterns in senescent annulus cells, and in how these patterns differed from those in non-senescent cells.

Table 4 summarizes significantly different expression patterns for genes related to extracellular matrix (ECM) formation and degradation, expression of growth factors and genes related to inflammation, genes related to cells signaling, and those to apoptosis.

Fibronectin type III and keratin 79, keratin associated protein 4-11, thrombospondin type I, domain contain 4, and spondin 1 (an ECM protein) were downregulated in senescent cells. Two matrix metalloproteinase were upregulated (MMP2 and an MIFR1), whereas ADAM metallopeptidase domain 3A was significantly downregulated.

Two genes related to fibroblast growth factor (FGF) showed significant differences in senescent cells (FGF % was downregulated, and FGF-receptor 2 was up-regulated). Significant upregulation was seen for bone morphogenetic protein-2 inducible kinase and interleukin 17C. Interleukin 25 and nitric oxide synthase 1 were downregulated.

Three important genes related to cell signaling showed significant downregulation in senescent cells: Mitogen-activated protein kinase 8 interacting protein 2, mitogen-activated protein kinase kinase kinase 11, and mitogen-activated protein kinase 2. Two other cell signaling genes showed significant upregulation in senescent cells: mitogen activated protein kinase kinase kinase 10, and cirhin.

Senescent cells showed significant downregulation of three genes related to apoptosis: BCL2/adenovirus E1B interacting proteins 2 and 3, and apoptotic peptidase activating factor 1.

Significant changes were also present in senescent cells in a number of genes related to solute transport, ribosomal proteins, zinc finger proteins, and other genes (Additional file 1: Table S1). Aquaporin 6 and ATG4 autophagy related 4 homolog B were significantly downregulated in senescent cells.

## Discussion

In this study we utilized LCM to separately harvest senescent and non-senescent cells in paraffin-embedded section of human annulus tissue from the intervertebral discs. LCM harvests produced mRNA in amounts which could then be utilized in whole genome microarray analysis. This application of LCM to selectively isolate senescent cells was especially important in our work because this is the only methodology whereby senescence cells can currently be separated from non-senescent cells in tissue. Researchers who are experienced with harvest of individual cells using laser capture microdissection will be able to carry out studies such as ours since senescent cells were readily visualized with the fluorescent microscopy as illustrated in Figure 1. We look forward to future LCM studies which also include real-time RT-PCR analysis of genes identified in our present work.

There is a known loss of cells in the aging and degenerating human disc; understanding senescent cells is important since their presence further reduces the disc's ability to generate new cells to replace those lost to necrosis or apoptosis.

Senescent cells, compared to those which were non-senescent, showed significant upregulation of a number of critical genes which have previously been shown to play important roles in cell senescence (Table 2).

Our analysis showed that mitogen-activated protein kinase p38 (also know as p38, p38 MPA kinase or MPAK14) was significantly upregulated in senescent compared to non-senescent annulus cells. Mitogen-activated protein kinase p38 plays an important causative role in senescent cells following telomere shortening [33-35]. Although we were not able to assess telomere length in the present work, important previous studies by LeMaitre et al [18] documented telomere shortening in cultured cells derived from degenerating disc specimens.

We found that senescent annulus cells showed significant upregulation of the gene growth arrest and DNA-damage-inducible beta (also called GADD45beta) compared to non-senescent cells. This gene is an upstream activator of the p38MAPK cascade (see above) [36]. Overexpression of GADD45beta has been shown to activate p38 via MTK1 [36-38]. Thus the GADD45beta gene also contributes to regulating the cell cycle.

Our analysis identified significant upregulation of the retinoblastoma (Rb)-associated KRAB repressor gene (also called RBAK) in senescent annulus cells compared to levels in non-senescent cells. A number of studies have previously shown that the retinoblastoma protein enforces permanent cell cycle withdrawal and that this gene plays a central role in senescence [39,40]. Additional studies have shown that the retinoblastoma-associated KRAB repressor gene contributes to Rb-dependent of E2F mediated transcriptional activation and Rb-mediated cell cycle arrest [41].

We found that discoidin, CUB and LCCL domain containing protein 2 was significantly upregulated in senescent annulus cells compared to non-senescent cells. Studies in vitro using 293T endothelial vascular cells, have shown that expression of this gene caused suppression of cell division [42].

Senescent disc cells expressed significantly greater levels of the gene inhibitor of growth family member 5 than did non-senescent cells. Members of the inhibitor of growth gene family are tumor suppressors which regulate cell cycle progression and also apoptosis and DNA repair; they are important cofactors of p53 [43].

Binding of somatostatin to its receptor has been shown to initiate G-protein-dependent cell growth arrest [44]. Our evaluation showed significantly greater expression of somatostatin receptor 3 in senescent annulus cells compared to non-senescent cells.

Interferon-induced transmembrane protein 1 has an important role in the anti-proliferative effects of interferon-gamma [45]. Yang et al. have proposed that it has its action by inhibiting extracellular signal-regulated kinase, enhancing the transcriptional activity of p53, and inhibiting p53 phosphorylation. In our work, we found that senescent cells had significantly great expression of interferon-induced transmembrane protein 1 compared to non-senescent cells.

Sphingosine 1-phosphate is a serum-borne bioactive sphingolipid which has been shown by Estrada et al. to have signaling functions in cells [46]. The receptor subtypes 1-3 for sphinogosine 1-phosphate are also known to be markedly increased in senescent endothelial cells, and Estrada et al. have shown that senescence can be blocked when the receptors are knocked down with molecular techniques. An series of studies have now shown that sphingolipids may have an important role in regulating cell senescence [47-49]. Our studies showed significant upregulation of sphingosine 1-phosphate receptor 2 in senescent vs. non-senescent cells.

Altered gene expression by senescent cells is now known to influence the surrounding microenvironment. Nitric oxide is a good example of an important mediation [50]. In studies by Sato et al. nitric oxide was shown to be inhibited by senescent endothelial cells [51], and decreased endothelial nitric oxide synthase mRNA, protein and activity was found to be greater in senescent endothelial cells compared to young cells [52]. Differential expression of nitric oxide synthase (eNOS) in senescent endothelial cells has also been identified by Bernardini et al. [53]. In our studies, we also identified a significant decrease in nitric oxide synthase in our senescent annulus cells compared to the non-senescent cells.

The p53 network, which is very complex, controls stress responses such as cell cycle arrest. P21 is a key mediator of p53-dependent cell cycle arrest. Cyclin-dependent kinase 8 (CDK8) acts as a stimulus-specific positive coregulator within the p53 system [54]. Recent work by Firestein et al. has shown that suppression of CDK8 expression inhibits proliferation in colon cancer cells [55]. In our analyses, we found a significant downregulation of CDK8 in senescent annulus cells compared to non-senescent cells.

The group of heat shock proteins (HSP) are stress-induced, and have a number of members which are now recognized to be associated with cell senescence, including HSP70 which was shown in the present study to be significantly downregulated in senescent annulus cells compared to non-senescent cells. Gutsmann-Conrad et al. have shown that the expression of HSP70 also decreased with senescence in IMR-90 lung fibroblasts during in vitro studies of cells from young or old subjects [56] and in senescent human fibroblasts [57]. In our analyses, we found a significantly reduced expression of HSP70 protein 6. This finding is important since this modification of the senescent cell phenotype may alter the cells ability to withstand hyperthermia and other different types of physiologic stresses.

Tables 3 and 4 present other genes important to disc cell function which showed significant differences in senescent vs. non-senescent annulus cells. Of these, it is interesting to note that with aging senescence-accelerated mouse (SAM) model shows a change similar to our finding with respect to the bone morphogenetic protein type II receptor. Takae et al. found that with the progression of degeneration this receptor could be found within annulus cells in this mouse model [58].

In closing, we would like to speak to the relevance and potential future applications of the findings presented here. Disc degeneration, and its associated low back pain, are a primary cause of disability and play a major role in this country's medical, social and economic structure. Estimated costs related to low back disorders are in the range of $50-100 billion per year in the U.S. alone [59]. Whether senescent cells are present in the disc as a result of disc degeneration, as a result of aging, or are due to as yet unidentified causes, it is now well-recognized that senescent cells are a important component of the disc cell population. A number of recent studies have shown that cellular senescence can be reversed, or at least attenuated [29,60-64]. Along with other researchers in the disc field, we feel that potential biologic therapies for disc degeneration hold much promise; information gained in the present study may one day contribute to future approaches which include anti-senescence therapies.

## Conclusions

The novel data presented here contribute to the understanding of senescence in the aging/degenerating disc. Since senescent cells have lost of the ability to divide, this further compounds the degenerative process in the disc. In addition, these senescent cells may be exerting an influence upon the surrounding microenvironment and nearby cells. Prominent in our findings of senescent vs non-senescent in vivo gene expression patterns in human annulus cells were senescence genes related to the pRB/p53 and MAP kinase pathways. Our application of LCM was a critically important experimental technique which allowed us to separately analyze senescent vs non-senescent cells. Major genes were identified which have recognized relationships to cell senescence, and gene-interactions within cellular senescence changes. Findings reported here may contribute to future biologic therapies for disc degeneration which include approaches to prevent cellular senescence.

## List of Abbreviations

ECM: extracellular matrix; FGF: fibroblast growth factor; HSP: heat shock protein; LCM: laser capture microdissection; L: lumbar; S: sacral; C: cervical.

## Competing interests

The authors declare that they have no competing interests.

## Authors' contributions

The study was conceived and planned by HEG in collaboration with ENH. JAL and NZ performed the slide preparation and carried out LCM cell harvests. GLH performed RNA isolations, specimens preparations for microarray analysis, and statistical analysis of microarray data with HEG. HEG wrote the manuscript assisted by GLH. All authors read and approved the final manuscript.

## Supplementary Material

Additional file 1 **Additional Genes with Significant Differential Expression Compared to Non-Senescent Annulus Cells**. This table provides additional supplementary information for the reader. Click here for file
