# Supplementary material for: Senescent vs. non-senescent cells in the human annulus in vivo: Cell harvest with laser capture microdissection and gene expression studies with microarray analysis
Source: BMC Biotechnol. 2010 Jan 28;10:5. doi: 10.1186/1472-6750-10-5 (PMC2828399; doi:10.1186/1472-6750-10-5)
Supplement: Additional file 1 — Additional Genes with Significant Differential Expression Compared to Non-Senescent Annulus Cells. This table provides additional supplementary information for the reader. [file 1472-6750-10-5-S1.DOC]

**Table S1. Additional Genes with Significant Differential Expression**

**Compared to Non-Senescent Annulus Cells**

| **Gene Name** | **Ratio/Fold**  **Change** | **Direction** | **P value** | **Gene Identifier** |
| --- | --- | --- | --- | --- |
| ***Extracellular Matrix:*** |  |  |  |  |
| Fibronectin type III and SPRY domain containing 2 | 1.26 | Up | 0.032 | AI375128 |
| Keratin 79 | 1.53 | Up | 0.026 | BC039148 |
| Keratin associated protein 4-11 | 1.05 | Down | 0.043 | AJ406944 |
| Thrombospondin, type I, domain containing 4 | 1.2 | Down | 0.017 | BG163478 |
| Spondin 1, extracellular matrix protein | 1.13 | Down | 0.008 | AI885290 |
| Galectin-related protein | 1.13 | Down | 0.045 | NM_014181 |
|  |  |  |  |  |
| ***Extracellular Matrix Degradation:*** |  |  |  |  |
| ADAM metallopeptidase domain 3A (cyritestin 1) | 1.51 | Down | 0.041 | X89657 |
| Alternatively spliced gene for Matrix Metalloproteinase in the Female Reproductive tract MIFR1, -2, MMP2122A, -B and -C (alternatively spliced CDC2L2 gene) | 1.24 | Up | 0.031 | AL031282 |
| Matrix metallopeptidase 2 (gelatinase A, 72kDa gelatinase, 72kDa type IV collagenase) | 1.22 | Up | 0.028 | AK057680 |
|  |  |  |  |  |
| ***Growth Factor- or Inflammation-Related:*** |  |  |  |  |
| Fibroblast growth factor 5 | 1.07 | Down | 0.022 | NM_004464 |
| Fibroblast growth factor receptor 2 (keratinocyte growth factor receptor) | 1.08 | Up | 0.035 | M80634 |
| Interleukin 25 | 1.08 | Down | 0.040 | NM_022789 |
| Interleukin 17C | 1.09 | Up | 0.030 | AF152099 |
| BMP2 inducible kinase | 1.16 | Up | 0.040 | AU144829 |
| Interferon, alpha 7 | 1.2 | Up | 0.042 | NM_021057 |
| Tumor necrosis factor (ligand) superfamily, member 8 | 1.07 | Up | 0.023 | AW518486 |
| Tumor necrosis factor receptor related protein mRNA, complete exon and repeat region (clone NCD18) | 1.17 | Down | 0.007 | L04489 |
| Tumor necrosis factor (ligand) superfamily, member 13b | 1.13 | Down | 0.024 | AF134715 |
| Bone morphogenetic protein receptor, type II (serine/threonine kinase) | 1.15 | Up | 0.023 | U20165 |
|  |  |  |  |  |
| ***Cell Signaling:*** |  |  |  |  |
| Mitogen-activated protein kinase 8 interacting protein 2 | 1.28 | Down | 0.042 | NM_016431 |
| Mitogen-activated protein kinase kinase kinase 10 | 1.41 | Up | 0.036 | NM_002446 |
| Cirhin | 1.57 | Up | 0.046 | AL578336 |
| Mitogen-activated protein kinase kinase kinase 11 | 1.08 | Down | 0.015 | NM_002419 |
| Mitogen-activated protein kinase kinase 2 | 1.18 | Down | 0.013 | AI762811 |
| RAS guanyl releasing protein 4 | 1.15 | Up | 0.014 | AA923524 |
| PTPRF interacting protein, binding protein 1 (liprin beta 1) | 1.17 | Up | 0.023 | AI962377 |
|  |  |  |  |  |
| ***Apoptosis-Related:*** |  |  |  |  |
| BCL2/adenovirus E1B 19kDa interacting protein 3 | 1.08 | Down | 0.037 | U15174 |
| BCL2/adenovirus E1B 19kDa interacting protein 2 | 1.12 | Down | 0.028 | BC002461 |
| Apoptotic peptidase activating factor 1 | 1.11 | Down | 0.031 | AF248734 |
|  |  |  |  |  |
| ***Solute Carrier-Related:*** |  |  |  |  |
| Solute carrier family 28 (sodium-coupled nucleoside transporter), member 1 | 1.22 | Down | 0.03 | AI206039 |
| Solute carrier family 29 (nucleoside transporters), member 2 | 1.24 | Down | 0.011 | BM709337 |
| Solute carrier family 35, member E1 | 1.76 | Down | 0.046 | NM_024881 |
| Solute carrier family 38, member 10 | 1.34 | Up | 0.048 | BF663461 |
| Solute carrier family 44, member 1 | 1.33 | Up | 0.042 | AK022549 |
| Solute carrier family 6 (neurotransmitter transporter, creatine), member 8 | 1.44 | Up | 0.012 | AI820043 |
| Solute carrier family 2 (facilitated glucose/fructose transporter), member 5 | 1.28 | Up | 0.042 | BE560461 |
|  |  |  |  |  |
| ***Calcium channel:*** |  |  |  |  |
| Calcium channel, voltage-dependent, T type, alpha 1I subunit | 1.29 | Up | 0.007 | AF211189 |
|  |  |  |  |  |
| ***Aquaporins:*** |  |  |  |  |
| aquaporin 6, kidney specific | 1.15 | Down | 0.017 | AL137716 |
|  |  |  |  |  |
| ***Integrins/Cell Adhesion:*** |  |  |  |  |
| Integrin, alpha 10 | 1.45 | Up | 0.01 | AF112345 |
| Integrin, beta 6 | 1.13 | Down | 0.049 | AA609987 |
| Integrin, alpha 6 | 1.11 | Up | 0.048 | AV733308 |
| Integrin-linked kinase-2 | 1.19 | Down | 0.039 | NM_004517 |
| Thrombospondin 4 | 1.11 | Down | 0.048 | NM_003248 |
| Alpha-2-glycoprotein 1, zinc-binding | 1.23 | Down | 0.041 | D90427 |
| Intercellular adhesion molecule 4 (Landsteiner-Wiener blood group) | 1.34 | Down | 0.04 | NM_001544 |
| Nidogen 1 | 1.12 | Up | 0.046 | NM_002508 |
| Vinculin | 1.06 | Down | 0.025 | NM_014000 |
|  |  |  |  |  |
| ***Ribosomal Proteins:*** |  |  |  |  |
| ribosomal protein L7a pseudogene 1 | 1.73 | Up | 0.038 | AJ224080 |
| Ribosomal protein L9 | 1.25 | Down | 0.019 | NM_000661 |
| ribosomal protein S14 | 1.45 | Up | 0.039 | AF116710 |
| Ribosomal protein S15 | 1.53 | Up | 0.037 | NM_001018 |
|  |  |  |  |  |
| ***Zinc Finger Proteins:*** |  |  |  |  |
| Zinc finger protein 224 | 1.21 | Down | 0.016 | BE464105 |
| Zinc finger protein 37A | 1.3 | Up | 0.037 | AU118165 |
| Zinc finger protein 545 | 5.55 | Down | 0.008 | AA927918 |
| Zinc finger protein 654 | 1.21 | Down | 0.037 | NM_018293 |
| Alpha-2-glycoprotein 1, zinc-binding | 1.23 | Down | 0.041 | D90427 |
|  |  |  |  |  |
| ***Autophagy or Peroxisome related:*** |  |  |  |  |
| ATG4 autophagy related 4 homolog B (S. cerevisiae) | 1.29 | Down | 0.029 | NM_013325 |
| Peroxisomal biogenesis factor 3 | 1.21 | Up | 0.040 | AU157140 |
|  |  |  |  |  |
| ***Other Genes:*** |  |  |  |  |
| Thyroid hormone receptor associated protein 2 | 7.58 | Down | 0.012 | AI862062 |
| IQ motif containing C | 12.83 | Down | 0.002 | NM_018134 |
| MAGI family member, X-linked | 5.14 | Down | 0.002 | NM_024859 |
| Similar to cis-Golgi matrix protein GM130 | 5.95 | Down | 0.004 | AF316855 |
| Golgi autoantigen, golgin subfamily a, 2 | 1.08 | Up | 0.045 | NM_004486 |
| Pyrin and HIN domain family, member 1 | 1.58 | Down | 0.005 | AK024890 |
| Misshapen-like kinase 1 (zebrafish) | 1.37 | Up | 0.029 | AF218033 |
